# Tuning Titanium Surface Properties via μPPEO for Improved Osseointegration and Cell Adhesion

**DOI:** 10.3390/ma18163792

**Published:** 2025-08-13

**Authors:** Natália Z. P. De Melo, Stephany C. F. Bessa, Jussier O. Vitoriano, Carlos E. B. Moura, Rodrigo S. Pessoa, Clodomiro Alves-Junior

**Affiliations:** 1 Programa de Pós-Graduação em Ciência da Saúde, Universidade Federal do Rio Grande do Norte, Natal 59012-570, RN, Brazil; nataliazpm@hotmail.com (N.Z.P.D.M.); ster.figueiredo@hotmail.com (S.C.F.B.); 2 Programa de Pós-Graduação em Ciência e Engenharia de Materiais, Universidade Federal Rural do Semi-Árido, Mossoró 59625-900, RN, Brazil; ssier_6@hotmail.com; 3 Department of Animal Sciences, Universidade Federal Rural do Semi-Árido, Av. Francisco Mota, 572, Costa e Silva, Mossoró 59625-900, RN, Brazil; carlos.moura@ufersa.edu.br; 4 Laboratório de Plasmas e Processos, Instituto Tecnológico de Aeronáutica, São José dos Campos 12228-900, SP, Brazil; rspessoa@ita.br

**Keywords:** porous surface, dental implants, PEO, cell adhesion, bioactive surface, titanium

## Abstract

This study investigates a novel approach based on micro-pulse plasma electrolytic oxidation (μPPEO), aiming to improve the control over key parameters such as the Ca/P ratio, the formation of anatase and rutile phases, and the porosity of titanium surfaces—factors that are critical for enhancing bioactivity. By employing electrical micro-pulses with widths of 50 μs or 100 μs, our aim was to restrict the discharge time and subsequent surface/electrolyte reactions. The results demonstrate that μPPEO-treated surfaces exhibit uniform pore diameters, a Ca/P ratio of approximately 1.67, and the better control of anatase/rutile formation. The μPPEO treatment successfully produced hydrophilic surfaces, with the 6Ti50 sample displaying the highest polar component of surface energy. Notably, this sample was the only one to support cell viability comparable to that of the polystyrene surface on the 24-well plate, emphasizing its strong potential for clinical applications. Across all treated surfaces, OFCOL osteoblasts displayed a spindle-like morphology with elongated filopodia, suggesting favorable cell interactions and adaptability to the treated surfaces. This study underscores the promise of PPEO as a valuable technique for biomedical applications, particularly in controlling and optimizing dental implant surfaces.

## 1. Introduction

Titanium implants are a safe and predictable alternative for surgical rehabilitation treatments [1]. In general, long-term failure rates with primary implantation are relatively low, occurring in a small percentage of patients (one to two percent) [2]. However, the demographic trend in industrialized countries is leading to an increase in elderly patients with clinical conditions such as compromised bone quality or quantity, and challenging comorbidities like diabetes mellitus, osteoporosis, bisphosphonate medication, or post-radiotherapy effects [3,4]. Thus, research in recent years has significantly focused on surface treatments, with the aim of improving the osseointegration process and reducing the risk of implant failure in these patients [4,5].

Effective osseointegration depends on the implant’s characteristics such as the macroscopic and microscopic topography, chemical composition, surface properties, and its chemical–biological interaction with bone tissue [6]. When appropriately designed, these properties facilitate bone–implant interactions. Ionic adsorption, protein absorption, communication between cells and the implant surface, as well as signaling for cell differentiation are events that lead to the integration of the biomaterial–bone [1,2].

Multiple techniques are employed to enhance the topography and chemistry of implant surfaces. These methods encompass acid etching, anodic oxidation, blasting, fluoride treatment, and calcium phosphate coating [2,4,5]. Plasma electrolytic oxidation (PEO) is currently a well-developed electrochemical technique used to produce bioceramic coatings with improved adhesion on metal surfaces such as titanium, magnesium, aluminum, and zirconium [6,7,8]. The PEO process is an extension of traditional anodization that utilizes voltages of up to 600 V, promoting discharges in the electrolyte and/or metal surface, thus generating the important chemical reactions necessary for the formation of the coating. The mechanism of the process includes electrochemical oxidation and numerous events of fusion and crystallization at the discharge sites [9].

One of the primary benefits of PEO is the creation of a porous structure on the im-plant surface, with pore sizes ranging from 0.1 to 10 μm. This porous mesh promotes osteoblast attachment and enables a gradual transition from the implant surface to bone, facilitating integration [10].

Recent advancements in pulsed plasma electrolytic oxidation (PPEO) have shown that precise control over the microstructure, crystalline phase, porosity, and topography can be achieved by tailoring the pulse widths to match the duration of electrical discharges [11,12,13]. Controlling the discharge duration in this manner minimizes unwanted side reactions and surface degradation, which are common in conventional oxidation techniques. Shorter pulse widths, for example, limit the surface’s exposure to high-energy discharges, reducing surface damage and enhancing pore uniformity. This precise control also enables the selective formation of specific crystalline phases, such as anatase and rutile, which are beneficial for bioactivity and osseointegration. Additionally, PPEO offers the opportunity to use calcium- and phosphorus-based electrolytes, producing bioactive surfaces with compounds like calcium phosphate and hydroxyapatite (HA) [12,14,15]. These calcium phosphate surfaces promote cell proliferation, osseointegration, and the formation of new bone tissue through osteoconduction without inducing toxicity [16,17]. Incorporating bioactive elements can significantly elevate the biological efficacy of materials, exemplified by strategies like the integration of HA particles through ion substitution [16], alongside addressing specific needs of the target surface, such as imparting antibacterial properties [17,18]. These elements additionally play a vital role in bolstering bone homeostasis, amplifying matrix mineralization, and fostering angiogenesis [19].

This study focuses on the development of titanium surfaces treated by μPPEO using pulse widths of 50 and 100 μs, with an emphasis on biomedical applications, particularly for dental implants. Key parameters such as the porosity, surface topography, Ca/P ratio, presence of rutile and anatase phases, wettability, and cellular response were carefully evaluated to determine the potential of μPPEO-treated surfaces for an enhanced bioactivity and clinical performance.

## 2. Materials and Methods

### 2.1. Sample Preparation

In this study, 40 commercially pure-grade 2 titanium discs procured from TiBrasil (São Paulo, Brazil), measuring 13 mm × 2 mm (diameter × thickness) were used. They were electrically connected to a conductive wire and embedded in resin so that only one disc surface was exposed to the treatment. The samples were submitted to metallographic preparation using water sandpaper with grit sizes of 220, 400, 600, 1500, and 2000. Then, they were polished using colloidal silica with hydrogen peroxide until they were 0.25 μm. The samples were washed with distilled water and dried using paper towels before being treated.

### 2.2. PEO Treatment

For the PEO process, the 40 titanium discs embedded in resin were divided into 5 groups, as described in Table 1, with one of the groups serving as the control where no plasma treatment was applied. In the experimental groups, the oxide layers were produced in an electrolytic cell as detailed in previous publications [12,18], consisting of a beaker containing 400 mL of electrolyte solution composed of 0.25 M calcium acetate monohydrate (Ca(CH_3_COO)_2_·H_2_O, Sigma-Aldrich, St. Louis, MO, USA, ≥99%) and 0.025 M sodium dihydrogen phosphate dihydrate (NaH_2_PO_4_·2H_2_O, Sigma-Aldrich, St. Louis, MO, USA, ≥98%), with a pH of 5.40.

The sample was anodically polarized in the cell. On the opposite side, at the cathode, a 50.0 mm × 2.0 mm stainless steel plate (length × thickness) was placed. A current of 0.5 A was applied using a pulsed power supply, Pulsa 6, Plasma LIITS^®^ (Campinas, SP, Brazil). The on-time (T_on_) and pulse repetition time (T_off_) were set to 50 or 100 μs for all experimental groups. The treatment time was 2 or 6 min (Table 1). The electrolyte solution was stirred at 500 rpm, while the temperature was controlled at 22 °C using a thermostatic bath.

### 2.3. Topographic and Chemical Analysis

The morphology of the coatings was determined based on the surface micrographs obtained using a high-resolution scanning electron microscope (SEM), (Vega3, Tescan, Brno, Czech Republic). The shape, size, quantity, and distribution of pores, as well as the coating thickness, were quantified using the ImageJ software 1.54p, which processed the electron microscopy images. Chemical analyses of the sample surface were conducted using an energy-dispersive X-ray spectrometer (EDS) (AztecLive, Oxford, Abingdon, UK) coupled to the SEM. In addition to the surface analysis, the Ca/P ratios were also determined at each position along a linescan.

### 2.4. Characterization of Crystalline Phase

X-ray diffraction analysis (XRD) was used to analyze the crystalline phases of the sample surface. A Shimadzu XRD-6000 X-ray diffractometer (Shimadzu, Tokyo, Japan) was used for this purpose, with Cu Ka radiation operating at 30 kV, at an angle ranging from 20° to 80°, with a scanning speed of 2° per minute, and a step size of 0.02°. The phases were analyzed using the High Score Plus software 5.2.

### 2.5. Wettability Test

The technique used was the determination of the static contact angle using the sessile drop method. The analysis was conducted using two samples for each experimental condition, with three measurements taken on each sample. The samples were positioned horizontally and a drop of 10 μL of distilled water was placed on them. The samples were dried before another drop was placed. Five drops were used for each sample. The same process was carried out using glycerol instead of distilled water to evaluate the polar and the dispersive components of the surface. The angle formed between the drop and the surface of the sample was determined. The lower this angle, the greater the wetting of the surface. A camera documented the shape of the drop at different moments and the contact angle was calculated after drop stabilization with the assistance of the Image J software (public domain). Surface free energy ($\gamma_{s}$) and its polar ($\gamma^{p}$) and dispersion ($\gamma^{d}$) components of the sample were determined from two sets of contact angles (water and glycerol) according to Owens–Wendt–Kaelble equation [20].

### 2.6. Cell Culture

Murine osteoblasts (OFCOL II, code 0192), obtained from the cell bank of the Federal University of Rio de Janeiro (BCRJ, Rio de Janeiro, Brazil) were cultured in a medium consisting of Dulbecco’s modified Eagle’s medium (DMEM) supplemented with 2 mM L-glutamine, 4500 mg/L glucose, 10% fetal bovine serum (FBS), and 1% penicillin–streptomycin. Cells were maintained at 37 °C in a humidified atmosphere with 5% CO_2_. The culture medium was changed every 3 days.

### 2.7. Cell Toxicology

The cell toxicology of OFCOL was assessed using the colorimetric in vitro assay Alamar Blue™ (Invitrogen, Life Technologies Corporation, Thermo Fisher Scientific, Waltham, MA, USA; DAL1100, Lot: 2120063). A total of 4 × 10^4^ cells were cultured for 24 h on plasma-treated surfaces (2Ti50, 6Ti50, 2Ti100, and 6Ti100), polished Ti, and 24-well plate surface. After incubation, cells were treated with Alamar Blue™ (1:10, reagent: DMEM) and incubated for 4 h at 37 °C with 5% CO_2_. Then, 100 µL of supernatant was transferred in triplicate to a 96-well plate and absorbance was measured at 570 nm (reduced form) and 600 nm (oxidized form) using a microplate reader (Epoch, Biotech company, Dover, MA, USA). Alamar Blue reduction was calculated following the manufacturer’s instructions.

### 2.8. Cell Morphology

To evaluate cell adhesion, 4 × 10^4^ cells/surface were added to the surfaces, followed by incubation for 4 h and then maintained at 37 °C in a humidified atmosphere with 5% CO_2_. After the incubation, the samples were washed with phosphate buffer saline (PBS) to remove nonadherent cells. Fixation was performed with 2.5% glutaraldehyde in 0.1 M phosphate buffer pH 7.4 (PBS) at room temperature for 2 h, followed by post-fixation with 1% osmium tetroxide in PBS for 2 h. Subsequently, the samples were washed with distilled water and dehydrated in a series of increasing ethanol concentrations (25, 50, and 75%) for 20 min each and in absolute ethanol for 60 min. After dehydration, the samples were coated with a 9 mm gold film (Q150R ES, Quorum Technologies Ltd., Laughton, East Sussex, UK) to enable visualization under a scanning electron microscope (SEM-SSX 550 Superscan, Shimadzu Corporation, Tokyo, Japan).

### 2.9. Statistical Analysis

Quintuplicate measurements were performed per surface in cell viability assay. Data were analyzed using statistical software (GraphPad Prism version 9.1.2) and found to be normally distributed (Shapiro-Wilk’s test). One-way ANOVA and Tukey’s multiple comparisons were used to compare cell viability for different surfaces. A significant level of *p* < 0.05 was considered.

## 3. Results

### 3.1. Topography, Chemical Analysis, and Crystalline Phases

**Figure 1:**
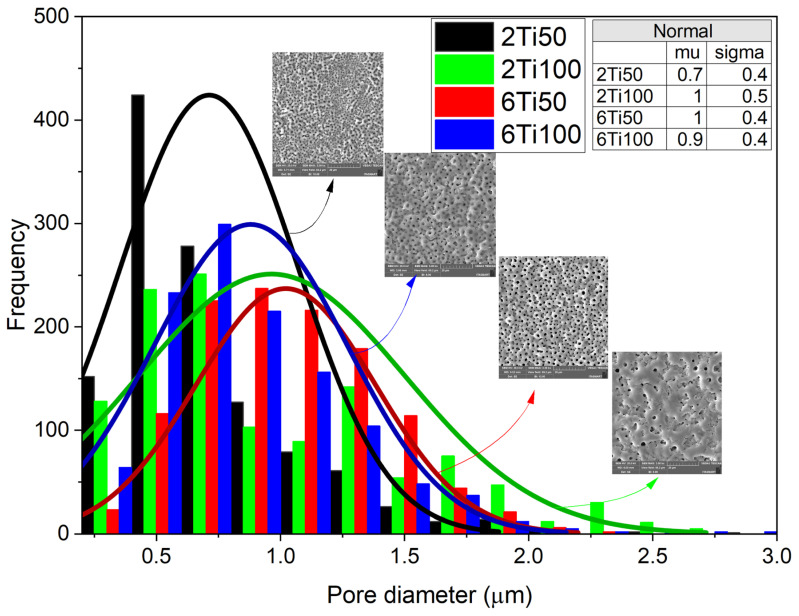
Histogram of pore diameter distributions for titanium surfaces treated with micro-pulsed plasma electrolytic oxidation under different conditions: 2Ti50, 2Ti100, 6Ti50, and 6Ti100. The colored bars represent the frequency of pores within specific diameter ranges for each treatment condition. Overlaid curves show the normal distribution fits for each condition, with parameters (mean, μ, and standard deviation, σ) provided in the inset table. Scanning electron microscopy (SEM) images of representative surfaces are included to illustrate pore morphology for each treatment condition.

Pore-size distribution curves provide valuable insight into both the size and uniformity of the pores generated during the process (Figure 1).

**Figure 2:**
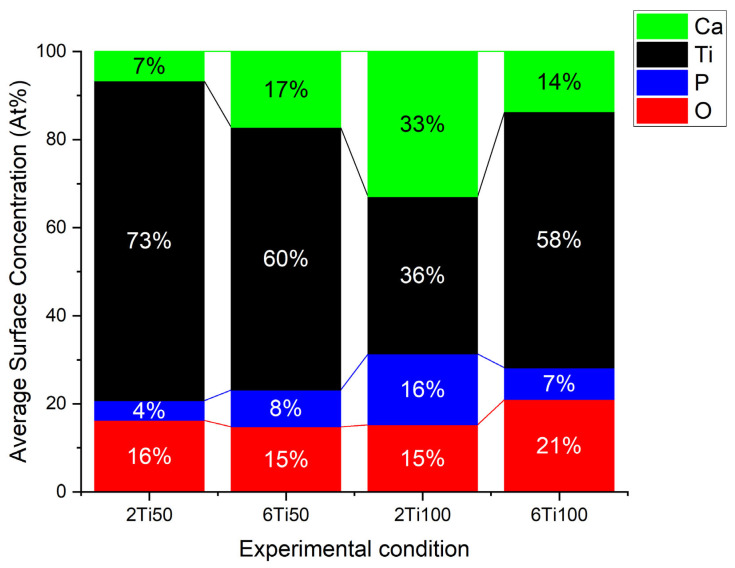
Average surface chemical concentration for titanium (Ti), oxygen (O), calcium (Ca), and phosphorus (P), obtained from line scan analysis of surface of titanium samples treated with micro-pulsed plasma electrolytic oxidation.

Electron micrographs, analyzed using image analysis software, reveal a clear dependence on the plasma parameters used, particularly the pulse width. In general, the pores are circular, with a median diameter between 0.7 and 1 μm. Sample 2Ti50, which was produced with a shorter treatment time and smaller pulse width, exhibits a lower diameter, showcasing a higher degree of uniformity compared to other samples. In this case, with the small pulse width, the micro-arcs on the sample surface transfer less energy per pulse, reducing the molten region because the shorter discharge duration limits heat accumulation. On the other hand, the 6Ti100 sample, which was produced with a longer treatment time and wider pulse width, exhibited the second smallest pore diameter. This apparent contradiction can be explained by the increased heat transfer per pulse, which causes partial pore closure due to the molten material generated during the action of the micro-arcs. This dynamic, associated with the discharge duration (T_on_) and the off-time interval (T_off_), also explains the variations observed in the average concentrations of surface elements. EDS (Energy Dispersive X-ray Spectroscopy) analysis was performed on representative regions of the different treated surfaces to determine the chemical composition and verify the distribution of the average concentration of elements on the surface (Figure 2). As observed, the surface elements primarily consist of titanium (Ti), oxygen (O), calcium (Ca), and phosphorus (P). The results highlight the differences in the elemental distribution between samples 2Ti50 and 2Ti100.

**Figure 3:**
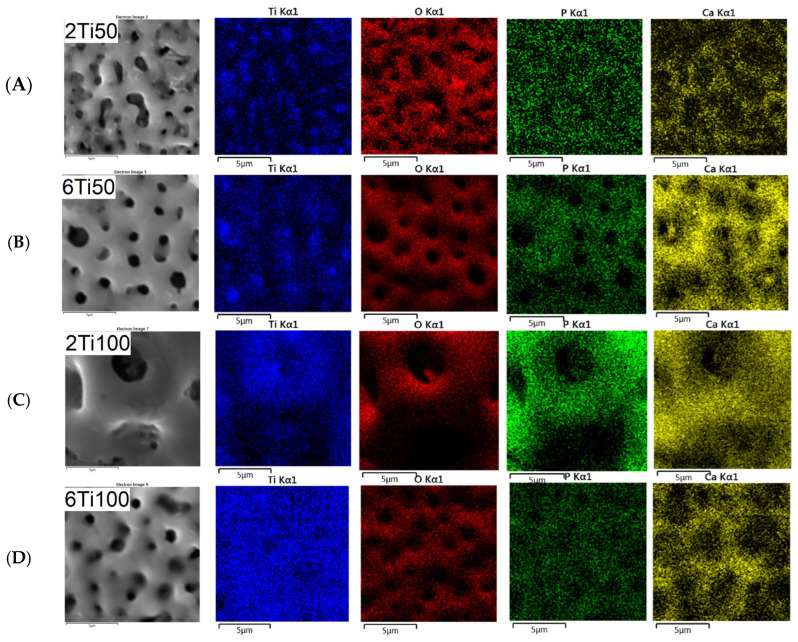
Line scan profiles of the Ca/P ratio across selected surface regions of μPPEO-treated titanium samples, indicating areas where the Ca/P ratio approaches the stoichiometric value of hydroxyapatite (1.67), as marked by the red dashed line. The Ca/P ratio is plotted along specific positions on the surfaces for each treatment condition: (**A**) 2Ti50, (**B**) 6Ti50, (**C**) 2Ti100, and (**D**) 6Ti100. Peaks above the red dashed line indicate localized regions where the Ca/P ratio is higher, suggesting the potential formation of calcium phosphate compounds. Variability across the line scans reflects the distribution and concentration of calcium and phosphorus on the treated surfaces.

A closer examination of the pore size distribution for sample 2Ti100 suggests the presence of a bimodal pattern with two local maxima—one near 0.6 μm and another around 1.4 μm. Although a unimodal normal distribution was used to fit all conditions in Figure 1 for comparative purposes, the 2Ti100 histogram deviates from this trend and indicates a more complex morphological profile. This feature likely results from the interaction of prolonged discharge durations (T_on_ = 100 μs) with the shorter treatment time (2 min), leading to both the formation and partial collapse of pores during micro-arc events. The heterogeneous pore structure observed in 2Ti100 may have contributed to the enhanced incorporation of calcium and phosphorus, as confirmed by the EDS maps in Figure 3. The increased surface area and capillary effects associated with bimodal porosity, combined with localized heating, likely promote ionic deposition and hydroxyapatite precursor formation during the PEO process.

Although both samples were treated for the same duration (2 min), the inversion of the discharge time (T_on_) and off-time (T_off_) led to significant variations in the surface composition. Ca^2+^ ions present in the electrolyte are primarily transported to the titanium surface by both electromigration and diffusion during the discharge events of the PEO process [14,15]. By adjusting the pulse duration ($T_{on}$) and pulse repetition time ($T_{off}$), precise control over the timing of microdischarges can be achieved, preventing excessive charge accumulation on the coating surface.

This approach allows for the better control of reactions occurring on both the electrolyte and titanium surfaces. A key factor influencing phase formation on the sample surface is the temperature and reaction time of the compounds, both of which are directly related to the pulse repetition duration. For the electrolyte used, the following reactions may occur [21]:

(1) $$Ca(COOCH_{3}{)}_{2}\to {Ca}^{2+}+2COOCH_{3}^{-}$$

(2) $$NaH_{2}PO_{4}^{-}\to {Na}^{+}+H_{2}PO_{4}^{-}$$

(3) $$H_{2}PO_{4}^{-}\to HPO_{2}^{2-}+H^{+}$$

(4) $$HPO_{4}^{2-}\to PO_{4}^{3-}+H^{+}$$

When the electrodes are polarized by a pulsed electric potential and subsequently turned off, the combined effects of ion migration under the external electric field and diffusion driven by the concentration gradient occur, initiating the following reactions to form hydroxyapatite [15]:

(5) $$10{Ca}^{2+}+6PO_{4}^{3-}+2OH^{-}\to {Ca}_{10}({PO}_{4}{)}_{6}(OH{)}_{2}$$

(6) $$10{Ca}^{2+}+6HPO_{4}^{2-}+{8OH}^{-}\to {Ca}_{10}(PO_{4}{)}_{6}(OH{)}_{2}+6H_{2}O$$

Although these reactions are commonly used to describe hydroxyapatite (HA) formation under alkaline conditions, the bulk pH of the electrolyte in this study was 5.4. Thus, the presence of excess OH^−^ in the bulk solution is unlikely. Instead, we propose that localized micro-arc discharges transiently increase the temperature and induce water dissociation near the titanium surface, generating reactive OH^−^ species in situ [22]. These localized regions may briefly reach elevated pH levels sufficient to promote the precipitation of calcium phosphate or HA-like compounds [23]. Therefore, Equations (5) and (6) are not representative of homogeneous reactions in the electrolyte, but rather of interfacial processes occurring in the high-field, thermally activated zones adjacent to the discharge sites [24]. This field-enhanced precipitation mechanism is supported by the observed Ca and P surface enrichment and should be further investigated through advanced structural or spectroscopic techniques. The extremely high localized temperatures (~1000–3000 °C) generated during the PEO process, combined with ultrafast cooling within microseconds, promote the formation of amorphous hydroxyapatite. Consequently, this phase frequently does not exhibit detectable peaks in X-ray diffraction (XRD) patterns [25]. While the electric field causes the separation of cations and anions, the opposite occurs when the pulse is turned off, allowing them to recombine. In the process of titanium dissolution near the sample (Equation (7)), oxygen can be generated, subsequently evolving as a gas (Equation (8)), or it can dissolve in the solution to generate TiO_2_ (Equation (9)) [21]:

(7) $$Ti\to {Ti}^{4+}+4e^{-}$$

(8) $$4OH^{-}\to 2H_{2}O+O↑+4e^{-}$$

(9) $${Ti}^{4+}+O^{2-}\to TiO_{2}$$

As the micro-pulsed voltage applied to the electrolyte increases, the thickness of the oxide layer progressively grows. When the anodizing voltage exceeds the dielectric breakdown threshold of the oxide film, weakened regions of the oxidation layer are disrupted by intense micro-arc discharges. Molten material is ejected from the discharge channels and solidifies upon contact with the surrounding solution. In the case of $T_{on}/T_{off}$ = 50/100, the micro-arcs and heating of the region are lower compared to $T_{on}/T_{off}$ = 100/50. This is due to the shorter duration of arc activity and the longer cooling period before the next pulse occurs. Additionally, the chemical gradient generated by the concentration differences, combined with the intense electric field (in pulsed mode), promotes the diffusion and incorporation of these species into the growing oxide layer [26]. Since the applied pulse is monopolar and the transport of Ca^2+^ occurs predominantly via a chemical gradient, it is suggested that a longer T_on_ and/or extended treatment duration may play a crucial role in enhancing ion migration toward the titanium surface. As shown in Figure 2, the calcium concentration increases with the treatment time when using a 50 μs pulse. However, when using a 100 μs pulse, the calcium concentration decreases with longer treatment times. A possible explanation for this seemingly contradictory result is the initial depletion of Ca^2+^ ions in the electrolyte during the first few minutes of treatment.

A Ca/P ratio of 1.67 was observed in several regions of the surface, suggesting the formation of hydroxyapatite, consistent with values reported in previous studies [27].

Unlike the Ca/P ratio, which had an average value close to the stoichiometry of hydroxyapatite, the O/Ti ratio was significantly lower than the stoichiometry of TiO_2_. This result is more indicative of the surface distribution of these elements than of the absence of stable oxide phases. Further analysis reveals a reduction in the oxygen concentration within the pores, indicating that these regions, despite experiencing elevated temperatures, do not undergo preferential oxidation (Figure 3). In contrast, the pores are predominantly composed of titanium (Ti). Additionally, the calcium profile closely mirrors that of phosphorus across all samples, suggesting a uniform co-deposition of calcium and phosphate species on the surface. This correlation indicates that the incorporation of these elements likely occurs through similar mechanisms during the plasma electrolytic oxidation process, which is consistent with the formation of calcium phosphate-based compounds. The localization of Ca and P primarily at the pore edges suggests that the pores remain open and unobstructed—an important characteristic for preserving bioactivity, enabling the ion exchange, and supporting cell interactions in biomedical applications. This distribution highlights the effectiveness of the process in ensuring uniform elemental incorporation while preserving pore functionality.

**Figure 4:**
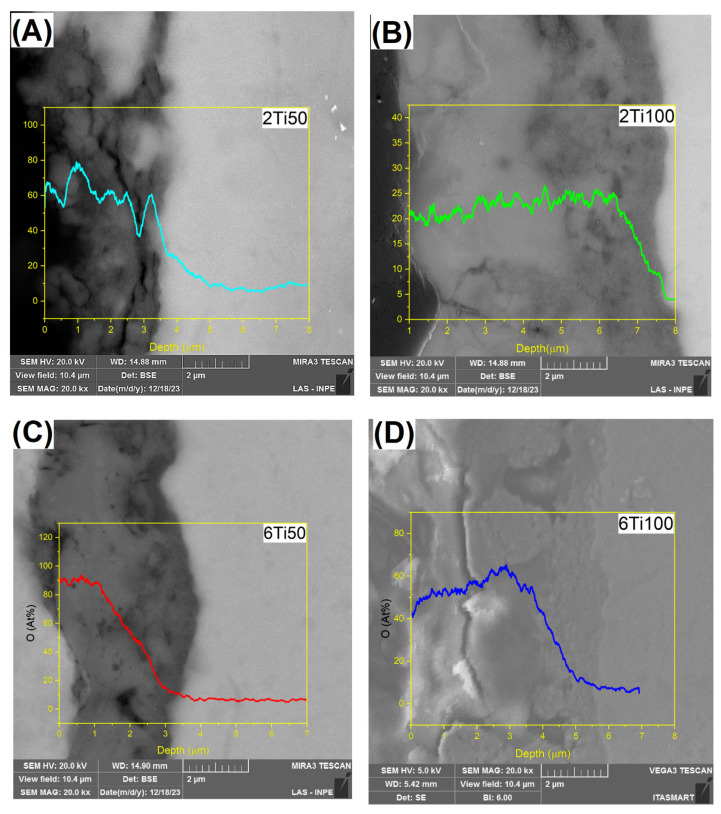
Depth profiles of oxygen concentration (at.%) for μPPEO-treated titanium samples, showing the variation of oxygen content with depth for different treatment conditions: (**A**) 2Ti50, (**B**) 2Ti100, (**C**) 6Ti50, and (**D**) 6Ti100. Each plot represents the oxygen concentration along the depth from the surface, with higher concentrations observed near the surface and a gradual decrease with depth. The images inserted in the background of each graph show corresponding cross-sectional views of the surface layers. Scale bars in SEM images represent 2 μm.

Upon analyzing the results obtained from EDS conducted on the sample layer depths, we observed the formation of a treatment-modified layer. The samples treated with a 50 μs pulse ON time consistently developed an oxide layer approximately 3.5 μm thick, regardless of the total treatment duration (Figure 4). However, the oxygen depth profiles varied. Samples treated for 6 min displayed a higher surface oxygen concentration, followed by a more abrupt decline with depth. A similar trend was observed in samples treated with 100 μs pulses. In this case, the oxide layer reached approximately 6.0 µm, but the 6 min treatment resulted in a steeper oxygen concentration gradient despite the higher surface enrichment. It is known in the literature that the thickness of the oxide layer varies with the treatment time, as indicated by various studies on oxidation processes in metallic materials [6,28,29]. This did not occur for this work, where the thickness remained constant at 3.5 μm and >6 μm for 2 and 6 min treatment times, respectively. However, the oxygen concentration throughout the layer in the 2Ti100 sample is higher than in the 6Ti100 sample. A plausible explanation for this unexpected result can be found by examining the general mechanism of oxide layer formation [29,30,31]. Initially, an oxide layer forms at the interface between the metal and the electrolyte, followed by an increase in the potential difference across the oxide, leading to the breakdown of the layer through discharge as the dielectric strength is exceeded [24].

At the start of the oxidation process, an outward thickening of the oxide layer occurs, followed by a slower inward thickening. This inward growth depends on the diffusion of oxygen ions through the pre-existing oxide layer. Once the outer layer stabilizes, oxygen diffusion becomes the key mechanism for further growth, thickening the oxide layer inwardly [21], specifically within the thermally affected zone.

**Figure 5:**
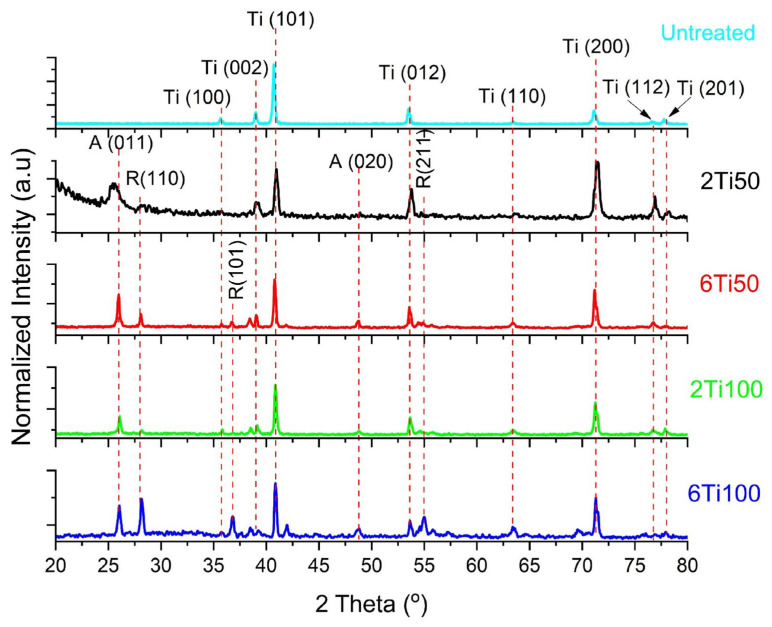
X-ray diffraction (XRD) patterns of μPPEO-treated titanium samples compared to the untreated titanium reference. The diffraction peaks indicate the presence of crystalline phases for each sample, with specific focus on anatase (A) and rutile (R) phases of titanium dioxide, as marked by red dashed lines. Peaks corresponding to titanium (Ti) are also labeled for clarity. The untreated sample shows prominent titanium peaks, while the μPPEO-treated samples (2Ti50, 6Ti50, 2Ti100, and 6Ti100) display varying intensities of anatase and rutile peaks depending on the treatment condition, reflecting the effects of μPPEO on phase composition. These variations in crystalline phase composition are relevant for understanding the bioactivity and potential clinical performance of the treated surfaces.

The X-ray diffraction analysis indicates the existence of both oxides in the treated samples (Figure 5). For the condition of the smallest pulse width, 50 µs, it is observed, by comparing peaks A (001) and R (110) of the anatase and rutile phases, respectively, that anatase is the predominant oxide phase. It is known from the literature that the anatase-to-rutile transformation occurs at temperatures between 450 °C and 850 °C [32]. In the case of PEO, it is estimated that this occurs when the electrolyte–film system heats up by approximately 6 °C [33]. Although the EDS analysis confirmed the presence of calcium and phosphorus on the surface, no peaks corresponding to crystalline Ca- or P-containing phases were detected in the XRD patterns.

Regarding the diffraction peaks at ≈24.8°, 38°, 42°, 56°–57.5°, 69.5°, and 76° observed for the 6Ti100 sample, we attribute these features to overlapping reflections of anatase and rutile TiO_2_ (e.g., anatase (002, 112, 224) and rutile (211, 301)) [34] combined with low-intensity signals from the α-Ti substrate. Notably, the peak at 24.8° may indicate trace amounts of amorphous or poor crystalline calcium phosphate within the surface layer. These additional peaks, which were absent in untreated or other μPPEO-treated samples, suggest subtle modifications in oxide crystallinity and potential minor Ca–P incorporation at the highest energy condition (6 min, 100 µs). While these did not manifest as discrete crystalline Ca–P phases, their presence aligns with the observed elemental enrichment and warrants further investigation to resolve these interfacial features. This suggests that these elements are incorporated in an amorphous form or are present in quantities below the detection limit of XRD.

### 3.2. Wettability Test

**Figure 6:**
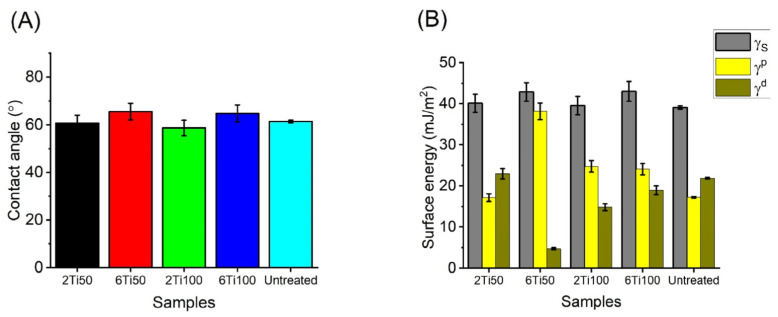
(**A**) Static contact angle measurements of distilled water on μPPEO-treated titanium surfaces and control samples, obtained using the sessile drop method. Error bars represent the standard deviation of measurements across multiple samples. (**B**) Surface energy analysis for each treatment condition, with total surface energy (γ_s_) decomposed into polar (γ^p^) and dispersive (γ^d^) components, derived from contact angles of distilled water and glycerol.

This study revealed that variations in the pulse ON time (T_on_) did not lead to significant differences in the contact angle measurements. The only parameter that influenced the wettability was the total treatment duration: samples treated for 2 min exhibited lower contact angles compared to those treated for 6 min (Figure 6). However, the 6Ti50 sample exhibited a significantly higher polar component, which is important for facilitating the adsorption of human osteoblast precursor proteins due to the increased negative charge density on the surface [35].

### 3.3. Cell Toxicology and Adhesion

**Figure 7:**
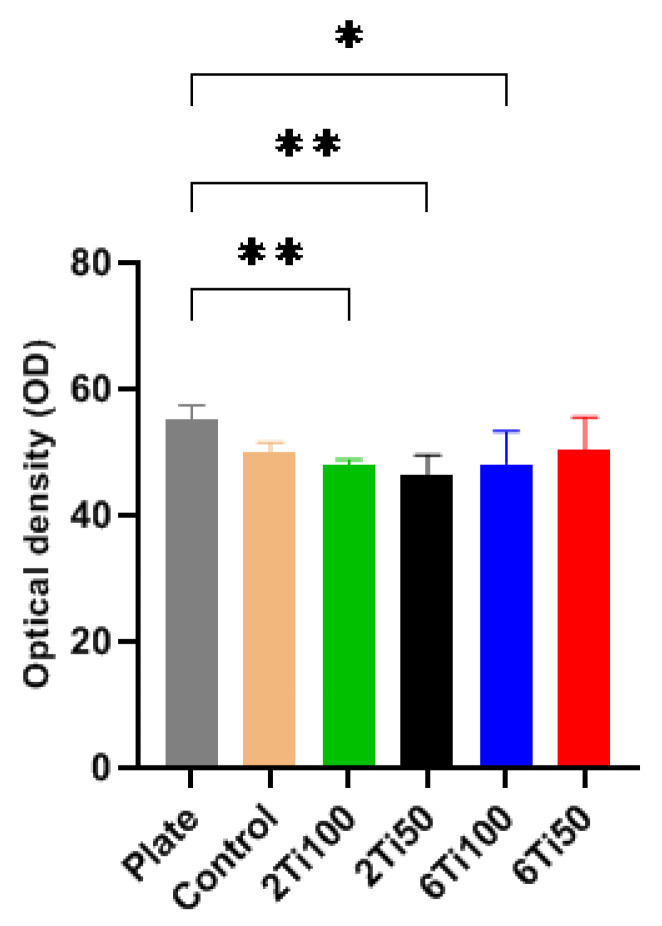
Optical density (OD) measurements of cell viability for μPPEO-treated titanium surfaces (2Ti100, 2Ti50, 6Ti100, and 6Ti50), the control titanium surface, and the polystyrene plate surface (plate). Bars represent the average OD values, with error bars indicating the standard deviation across quintuplicate measurements. The asterisks (*) indicate statistically significant differences, * *p* < 0.05, ** *p* < 0.01, compared to the polystyrene plate surface. The results show that only the 6Ti50 treatment achieved cell viability comparable to the plate surface, while other treatments showed lower viability levels.

Optical density (OD) measurements of cell viability within the first 24 h revealed that OFCOL cell viability was slightly reduced on both treated and polished titanium surfaces (Figure 7). This sample demonstrated cell viability comparable to that observed on the polystyrene surface of a standard 24-well culture plate, commonly used as a control in cell culture experiments [36].

The OFCOL II osteoblastic cell line is commonly used to assess the toxicity of new materials on bone cells [37]. Toxicology assays are typically conducted within the first 12–24 h of cell exposure, allowing the observation of immediate cellular responses before adaptation or recovery [38]. After 24 h, significant environmental changes can occur, such as cellular matrix production and potential inflammatory or immunological responses [39]. Therefore, the initial 24 h are ideal for evaluating early cellular responses and material cytotoxicity.

**Figure 8:**
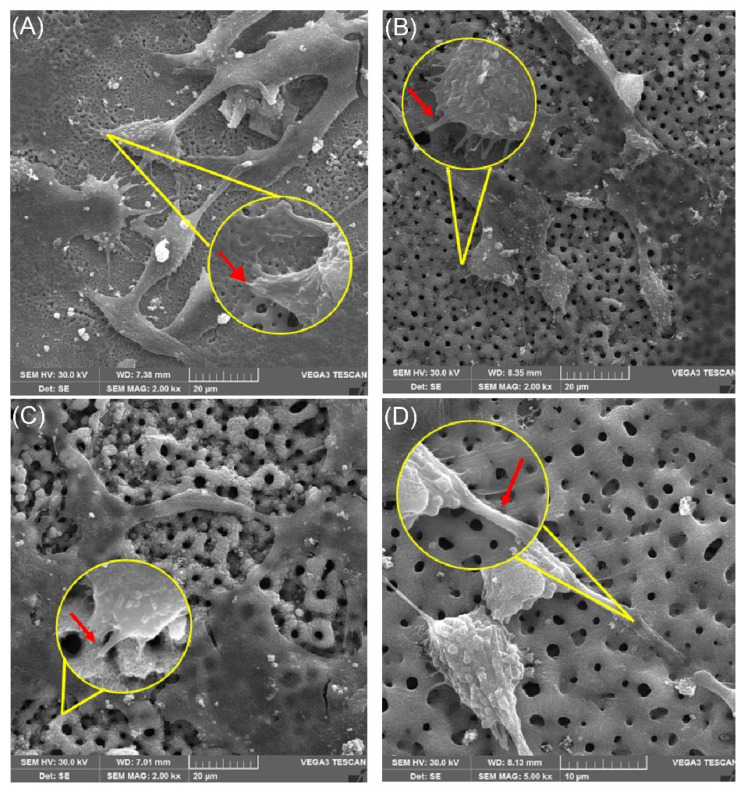
Scanning electron microscopy (SEM) images showing the interaction of OFCOL cells with titanium surfaces treated by micro-pulsed plasma electrolytic oxidation (μPPEO). Images captured at 2 kX magnification and 15 kX detail illustrate cell morphology and adhesion across samples: (**A**) 2Ti50, (**B**) 6Ti50, (**C**) 2Ti100, and (**D**) 6Ti100. Yellow circles highlight regions of interest where cells are in close contact with the surface. Red arrows indicate the presence of filopodia extensions anchoring the cells to the porous titanium structure, facilitating stable cell–surface interactions and promoting biointegration.

Although all surfaces exhibited low cytotoxicity, notable differences were observed between the control sample and the plasma-treated samples, particularly regarding the manner of cell–surface interactions. In the SEM evaluation, spindle-like cells with the emission of many long filopodia were observed on all treated surfaces, while on the polished surfaces (control), they presented a flattened morphology with few short filopodia (Figure 8)

These filopodia extensions promote favorable cell–matrix interactions, facilitating the recruitment of actin-mediated local forces to form stable focal adhesions [40,41]

**Figure 9:**
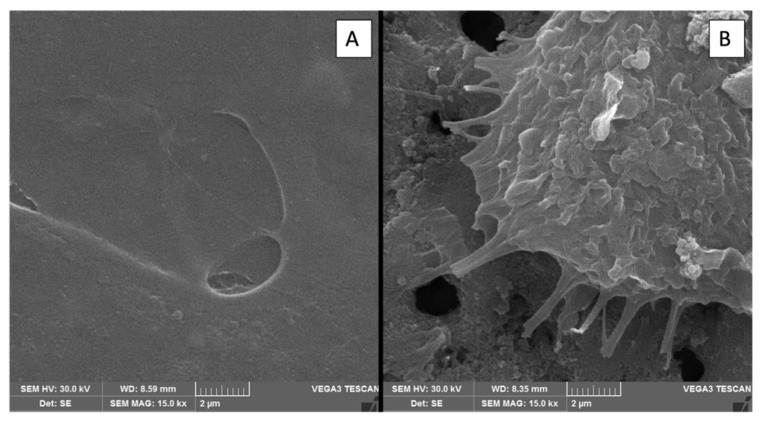
Scanning electron microscopy (SEM) images comparing cell morphology on titanium surfaces of the control group and μPPEO-treated group (6Ti50). (**A**) Cell on the surface of the control group, showing minimal cell spreading and attachment. (**B**) Cell on the surface of the experimental group (6Ti50), displaying extensive filopodia extensions that anchor the cell to the porous structure.

This suggests a better cell interaction with the surface, as demonstrated by the filopodia engaging with the pore edges, as shown in the 15 kX magnification in Figure 9B. In contrast, on the polished control surfaces, the cells appeared flattened with few filopodia (Figure 9A), indicating minimal anchorage to the surface.

## 4. Discussion

Previous studies have shown that polycarbonate membranes with pore diameters ranging from 0.2 µm to 1.0 µm resulted in increased adhesion and spreading of MG63 osteoblast-like cells, whereas pores with diameters of 3.0 µm to 8.0 µm exhibited spherical cell morphologies [42]. All our results have average pore diameter values below 1.5 µm. Therefore, despite the greater dispersion of the treated samples, they have candidate values for the good adhesion of osteoblastic cells.

When we talk about drug delivery systems, a porous and uniform structure is crucial. While the diameter of the surface pores is associated with the release rate, the volume is associated with the drug release time [43,44]. The growth rate of the titanium oxide layer in a PEO process depends, in general, only on the current density and treatment time [45]. Previous work conducted in our laboratory [46] showed that the growth rate of the titanium oxide layer, treated using the same electrolyte as in the present study, also varied with the T_on_/T_off_ ratio.

Both titanium oxide and calcium-phosphate (Ca-P) coatings are important for dental implant osseointegration. While the oxide layer has been shown to promote osteoblast attachment and functionality, the release of Ca-P, mainly composed of hydroxyapatite (Ca/P ratio = 1.67), into the peri-implant region increases the saturation of body fluids and precipitates a biological apatite onto the surface of the implant [47,48,49].

Comparing the processes carried out in 2 min, the sample treated with a 100 µs pulse significantly increased the amount of Ca and P deposited on the Ti surface, justifying the reduction in the average concentration of the titanium from 72 atomic percent to 34 atomic percent. The debate continues about the best Ca/P ratio to achieve greater osteoblastic differentiation [50]. On the other hand, samples treated for 6 min showed the same average concentration of Ti, indicating that the exposed surface is the same for both cases. The presence of these elements exclusively on the exposed surface indicates the efficiency of the process, as it ensures that the pores remain unobstructed and available to receive the drugs.

Regarding the oxides formed on the surface, both rutile and anatase have been shown to promote osteoblast attachment and functionality. However, the anatase phase can additionally inhibit bacterial adhesion [47,51]. Since the anatase phase is only stable at lower temperatures [33], this result can be justified by the lower energy incidence of this condition.

Not only the morphology and the composition of the titanium oxide may be modified by PEO, but a modification on the surface energy and wettability can be obtained and this can be an advantage for the cell behavior due to it being able to accelerate the recruitment of the cell adhesion protein and, after that, cell attachment [35]. All the groups had a satisfactory result and formed a hydrophilic surface. This characteristic is a fundamental point to the first steps of osseointegration such as protein adsorption. This is the case of those proteins that contain arginine-glycine-aspartate (RGD) which can have a direct effect on the cell attachment and integration of osteoblasts with the implant [8,9]. Other important elements for bone regeneration are the bone morphogenetic proteins (BMPs) in special BMP-2 and BMP-7 [52].

It is well-known how the roughness of titanium affects the wettability, protein adsorption on the surface, and surface energy in both the polar and dispersive components. These physicochemical properties are key factors for cell migration, proliferation, and differentiation [15]. The surface free energy of a solid is another fundamental parameter to be considered. It is constituted by the addition of its polar and dispersive components. The higher the non-polar or dispersive character of a surface, the more difficult adhesion is in general because it depends on the liquid on the surface. The opposite is true for a surface of a more polar character, which reflects hydrophilic interactions [53]. It is also well known that the titanium surface roughness affects the wettability, protein adsorption, and surface energy—both in its polar and dispersive components. These physicochemical properties play a crucial role in regulating cell migration, proliferation, and differentiation [20].

The mechanism of osseointegration is based on the interactions of the osteoblastic cells with the surface of the biomaterial and the generation of collagen scaffolds in which the apatite and other organic components will be deposited for the formation of the so-called bone matrix and, consequently, the formation of new bone tissue. We can assure that the adhesion of osteoblasts and their cellular activity together with the formation of the extracellular matrix of cells are key for the subsequent stages of proliferation and differentiation to complete the osseointegration process [54].

Based on this well-established knowledge from the literature on bioactive surfaces, Table 2 was constructed to summarize the influence of micro-pulse parameters (T_on_ and T_off_) on key surface characteristics. These include the pore size, Ca/P, O/Ti, contact angle, and Anatase/rutile—factors that collectively determine the bioactivity potential and suitability of the titanium surface for osseointegration and potential drug delivery applications.

The O/Ti and Ca/P ratios provide relevant insights into the presence of titanium oxides and hydroxyapatite on the surface, respectively, assuming the coexistence of these elements within the same analysis sites. An O/Ti ratio of two indicates the complete formation of TiO_2_ across the entire surface. As XRD results confirmed the exclusive presence of TiO_2_, the O/Ti values presented in the table—although lower than two—can be interpreted as an indicator of the extent of the surface area effectively covered by this oxide. In this context, it is observed that a pulse width of 100 μs favored a greater formation of oxide.

The lower anatase/rutile ratio observed in sample 6Ti100 can be attributed to increased solution heating, resulting from the combined effect of a longer pulse width and extended treatment time.

The Ca/P ratio, in turn, serves to assess how closely the composition of the surface layer approaches that of hydroxyapatite (Ca/P ≈ 1.67), which is critical for promoting bioactivity and osseointegration in biomedical applications. Values near this stoichiometric proportion suggest a higher potential for integration with bone tissue, thereby enhancing the coating’s effectiveness as a functional surface for implants.

Considering the standard deviations reported, all experimental conditions resulted in surfaces with bioactive potential. In other words, by adjusting the treatment time and pulse width, it is possible to fine-tune the formation of bioactive surfaces that are more favorable to successful osseointegration.

To endorse this result, we performed a SEM. The assessment of the cell morphology is widely used as a robust and direct indicator of surface biocompatibility, offering detailed insights into cell–surface interactions that are not captured by cellular metabolism-based colorimetric methods [55,56,57]. Cell morphology is determined as a function of the dynamic interactions and balance between the forces that occur between the cytoskeleton, the cell membrane, and the adhesion complexes that interact with the extracellular matrix often through the actions of cellular response regulatory transduction systems, such as migration, proliferation, and differentiation [58]. Therefore, morphology is a useful parameter for screening biomaterials [55].

In the present work, the evaluation of cellular adhesion analyzed in electromicrographs allowed us to verify anatomical characteristics more compatible with osseointegration, such as a fibroblastoid format (Figure 8) of the cells on the treated surfaces, with a greater number of filopodia. This may represent the better interaction of the cells on the surface, demonstrated by the interaction of the filopodia with the margins of the pores, as shown in Figure 9. Meanwhile, on the polished surfaces (control), the cells were flattened with few filopodia (Figure 9A), representing anchorage with the surface.

Meanwhile, cell proliferation can be activated by mechanotransduction pathways where features of surfaces such as the roughness, morphology, and surface tension are involved in an active way. Changes in the surface configuration can address the cellular response in some specific direction, for example, in the acceleration of osseointegration [12,14,15]. The presence of pores allowed for greater cell anchorage, while on the control surface (polished surface), the morphology achieved would compromise osseointegration.

The combined assessment of the pore morphology, elemental composition, crystalline phase distribution, and cell adhesion response validates the potential of μPPEO as a controllable and scalable method for enhancing the bioactivity of titanium-based im-plants. Future studies should expand upon this framework to include in vivo assessments and explore advanced drug loading strategies in relation to the surface chemistry and porosity.

## 5. Conclusions

This study demonstrated that micro-pulsed plasma electrolytic oxidation (μPPEO) enables the fine-tuned engineering of titanium surface properties through the systematic modulation of the pulse width and treatment time. By controlling these parameters, it was possible to tailor the pore morphology—including the emergence of bimodal distributions—alongside Ca/P ratios and the anatase/rutile phase composition. These features are critical for enhancing osseointegration and bioactivity. Among the tested conditions, 6Ti50 exhibited the most favorable response in terms of the wettability and cell adhesion, with clear filopodia extension toward pore edges. The formation of anatase-rich TiO_2_ and surface enrichment in Ca and P support the field-enhanced interfacial mechanism proposed for HA-like compound formation. These results establish μPPEO as a promising strategy not only for optimizing the biointegration of titanium-based implants, but also for enabling multifunctional surfaces suited to drug delivery and tissue regeneration applications.

## Figures and Tables

**Table 1:** Plasma electrolytic oxidation (PEO) treatment conditions for titanium samples. Sample names are designated based on treatment duration and pulse width. For example, “2Ti50” indicates a sample treated for 2 min with a pulse width of 50 μs. T_on_ and T_off_ represent the pulse-on and pulse-off times, respectively, in microseconds (μs). The control sample was untreated.

Sample	T_on_ (µs)	T_off_ (µs)	Treatment Time (min)
Control	-	-	-
2Ti50	50	100	2
6Ti50	50	100	6
2Ti100	100	50	2
6Ti100	100	50	6

**Table 2:** Relevant parameters for evaluating the bioactivity of titanium surfaces modified by micro-pulses.

Sample	O/Ti Ratio	Anatase/Rutile Ratio	Ca/P Ratio	Pore Size (μm)	Contact Angle (°)
2Ti50	0.2 ± 0.1	1.2	1.5 ± 1.0	0.7 ± 0.4	59 ± 3
2Ti100	0.4 ± 0.6	1.6	2.0 ± 1.3	1.0 ± 0.5	61 ± 3
6Ti50	0.3 ± 0.7	1.6	2.0 ± 1.5	1.0 ± 0.4	65 ± 4
6Ti100	0.4 ± 0.4	0.9	1.6 ± 1.0	0.9 ± 0.4	66 ± 3

## Data Availability

The original contributions presented in this study are included in the article. Further inquiries can be directed to the corresponding author.
